# Between living and aging: perceptions of women with HIV

**DOI:** 10.1590/0034-7167-2025-0084

**Published:** 2026-07-27

**Authors:** Evanilza Maria Marcelino, Jaciel Bezerra da Silva, Carla Andreia Alves de Andrade, Sergio Corrêa Marques, Aurélio Molina da Costa, Denize Cristina de Oliveira, Fátima Maria da Silva Abrão

**Affiliations:** I Universidade de Pernambuco. Recife, Pernambuco, Brazil; II Universidade Federal de Alagoas. Maceió, Alagoas, Brazil; III Universidade do Estado do Rio de Janeiro. Rio de Janeiro, Rio de Janeiro, Brazil

**Keywords:** Social Representation, Women, Healthy Aging, HIV, Nursing., Representación Social, Mujeres, Envejecimiento Saludable, VIH, Enfermería.

## Abstract

**Objectives:**

to analyze the perceptions of living and aging among women with HIV.

**Methods:**

qualitative research with 30 women aged 50 or older in Recife, PE, Brazil. The Social Representation Theory was used as a theoretical framework, and the lexical analysis method was employed using the Interface de R pour les Analyses Multidimensionnelles de Textes et de Questionnaires software.

**Results:**

the social representation of aging with HIV is viewed from the temporal perspective of a sad, painful, and revolting life trajectory, determining meanings about the diagnosis, daily life practices and activities, conflicts in human relationships, and adherence to antiretroviral therapies.

**Final Considerations:**

the importance of these representations lies in improving healthcare professionals’ practice and promoting a broader understanding of aging with HIV, offering more humanized, comprehensive/dynamic care in healthcare services, and considering quality of life in different social contexts.

## RESUMEN

**Objetivos:**

analizar las percepciones sobre la vida y el envejecimiento en mujeres con VIH.

**Métodos:**

investigación cualitativa con 30 mujeres de 50 años o más en Recife, PE, Brasil. Se utilizó la Teoría de las Representaciones Sociales como marco teórico y se empleó el método de análisis léxico mediante el *software Interface de R pour les Analyses Multidimensionnelles de Textes et de Questionnaires*.

**Resultados:**

la representación social del envejecimiento con VIH se contempla desde la perspectiva temporal de una trayectoria vital triste, dolorosa y repugnante, que determina los significados en torno al diagnóstico, las prácticas y actividades cotidianas, los conflictos en las relaciones humanas y la adherencia a las terapias antirretrovirales.

**Consideraciones Finales:**

la importancia de estas representaciones radica en mejorar la práctica de los profesionales de la salud y promover una comprensión más amplia del envejecimiento con VIH, ofreciendo una atención más humanizada, integral y dinámica en los servicios de salud, y considerando la calidad de vida en diferentes contextos sociales.

## INTRODUCTION

Time passes, but the constructs of social thought remain transient and variable in the 40 years since the discovery of AIDS in Brazil. Despite the success of antiretroviral therapies (ARTs), which increase life expectancy/quality of life and reduce AIDS cases, infections with the Human Immunodeficiency Virus (HIV) have not followed this same trend^(1)^.

In 2021, the global and national panorama was worrying, with 37.7 million people living with HIV worldwide, of whom 53% are women. There was an increase in the proportion of HIV cases among women aged 50 or older, from 12.2% in 2011 to 17.9% in 2021^(1,2)^.

Over the years, people who have contracted HIV are more likely to develop age-related diseases, directly impacting health indicators and their positive self-perception of living and aging with the virus^(2,3)^.

The Social Representation Theory (SRT) is relevant for understanding social phenomena. It aims to recognize and amplify the understanding of common sense, potentially influencing individual behavior. Moscovici, with his theory, developed processes that facilitate the exchange of information and the collective production of knowledge in communities in relation to a common object^(4,5)^.

According to Jodelet, social representations (SRs) interpret everyday reality based on knowledge shared between individuals and groups. They familiarize the new and the unusual within their representational universe, giving meaning to adopted behaviors. At the beginning of the discovery of AIDS, the visual elements of SRs were especially emphasized in the advertisements of time. This generated various social meanings, which today are confronted with visual representations of other social objects, such as female aging^(4-7)^.

The three dimensions present in SRs allow for a dimensional analysis: information (concept); the field of representation (image); and attitude. In this study, the concept is formed by the organization of knowledge of social subjects and the social object, varying according to the time, quantity, and quality of the knowledge disseminated^(8)^.

Reflecting on the subjectivity manifested in actions and reactions to the diagnosis, living with, and aging with HIV, women reveal SRs in their discourses. These SRs are understood as constructions influenced over time by historical and cultural origins, traditions, images, concepts, attitudes, and behaviors^(5-8)^.

This study is relevant considering the gaps found in the state-of-the-art review of the subject matter and the social group chosen for analysis. The production of SRT on HIV/AIDS is rarely discussed in national and international literature. However, it is very practical, given the actions and behaviors linked to living with HIV. This situation significantly impacts issues of gender, health, economy, and quality of life^(9-12)^.

This study aimed to gain a deeper understanding of the topic and consider the context of these women, proposing strategies for coping with life and aging while living with the virus.

## OBJECTIVES

To analyze the perceptions of living and aging among women with HIV.

## METHODS

### Ethical aspects

The established norms were observed in accordance with Resolution 466/2012, which provides guidelines and regulations for research involving human beings, with approval granted by a Research Ethics Committee. Participants were informed through the Informed Consent Form, which was subsequently signed in full or by means of a digital signature of their right thumb.

### Theoretical-methodological framework

This research was based on the procedural approach to SRs developed by Denise Jodelet in her study on SRs of madness^(13)^. The author understands SRs as socially constructed and shared knowledge with a practical objective, contributing to the construction of a common reality for a social group^(6)^.

The analysis of statements was carried out using the *Interface de R pour les Analyses Multidimensionnelles de Textes et de Questionnaires* (IRaMuTeQ) software, a tool that enables the systematic processing and organization of textual data. This resource allows for the identification of word frequency, the establishment of associations, and the classification of the material into categories, thus facilitating the understanding of the regularities and specificities present in the discourses^(14)^.

Applying this perspective to nursing, especially in the care of older women living with HIV, SRs become fundamental to understanding how these women perceive themselves, the disease, and treatment. SRs directly influence issues such as adherence to ART, seeking social support, coping with stigma, and how they deal with aging associated with HIV infection.

Furthermore, by studying these representations, nursing professionals can identify symbolic and cultural barriers that impact care, and develop more effective and humanized strategies. This also contributes to care that is more sensitive to the specific needs of this population, promoting a more critical, reflective, and person-centered practice.

### Study design

This is an exploratory-descriptive study with a qualitative approach. Semi-structured interviews were conducted, encouraging patients to share their perceptions about living and aging with HIV. The guideline adopted for preparing this study report was the COnsolidated criteria for REporting Qualitative research.

### Study setting

This study was conducted in two Specialized Care Services (In Portuguese, *Serviços de Assistência Especializada* - SAEs) for HIV/AIDS located in public hospitals in the Metropolitan Region of Recife, Pernambuco.

### Population

Thirty women, randomly but intentionally selected, participated in the study. All were heterosexual, aged 50 or older, and followed up at two SAEs. In addition, they had been diagnosed with HIV for at least six months. Women with cognitive impairment or mental disorders were excluded from this research.

### Data collection and organization

Data collection took place from June to August 2023, using a sociodemographic and clinical questionnaire, as well as a semi-structured interview guide to understand participants’ subjectivity. The themes of the guide focused on the field of representations, images, and knowledge constructed by the population about HIV, with the aim of understanding the attitudes and logic of social relations in relation to gender and aging.

The interviews, lasting an average of 11 minutes and 12 seconds, were audio-recorded with the participants’ permission, then transcribed and prepared for analysis. To preserve their anonymity, they were identified with the letter “W”, from the word “Woman”, along with the sequential number of the interview, age, and time since diagnosis (e.g., W1, age, time since diagnosis more than 20 years ago).

### Data analysis

Sociodemographic and clinical characterization data were consolidated in Microsoft Excel^®^ and analyzed using descriptive statistics. After organizing the textual database, it was entered and processed with the support of IRaMuTeQ, which provided a description of the content of participants’ statements. This software has been used in qualitative studies, mainly in studies of SRT, as it provides statistical techniques to help researchers understand the social object and create analytical classes based on the identification of units of meaning^(14)^.

Among the possible methods for analyzing the software, the Descending Hierarchical Classification method was chosen, in which each text was assessed using the material classification method, fragmented into parts and grouped according to words to construct categories.

### Declaration of generative AI and AI-assisted technologies in the drafting process

During the preparation of this manuscript, ChatGPT was used to assist in grammatical and spelling revision, as well as to generate suggestions for reformulating sections of the text. After using this tool, the content was reviewed and edited as necessary, with full responsibility assumed for the final content of the publication.

## RESULTS

The study included heterosexual women, with the majority (56.7%) aged between 50 and 59 years, and 46.7% living with HIV for more than 20 years. The majority (60%) identify as mixed-race and have incomplete primary education (56.7%). Regarding marital status, 36.7% are married and 36.7% are single. There is an equal proportion (40%) between retired and domestic workers, with 60% of them being Catholic.

The *corpus* was divided into 669 text segments. Of these, 586 text segments were processed, and after several cleavages, they stabilized and generated five lexical classes, with a retention rate of 87.59%. This percentage represents good material utilization, since, according to the literature, a minimum retention of 75% of the provided text segments is necessary^(14)^.

**Figure 1:**
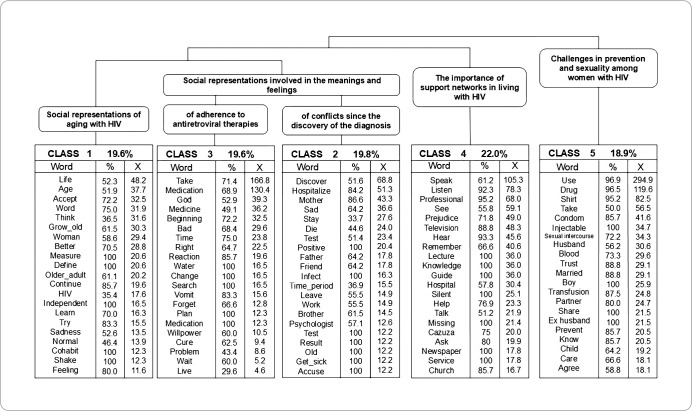
Dendrogram and Descending Hierarchical Classification of classes generated by IRaMuTeQ, Recife, Pernambuco, Brazil, 2025

The classes were defined according to Figure 1, as follows: Class 1 - Social representations of aging with HIV; Class 2 - Social representations involved in the meanings and feelings of adherence to antiretroviral therapies; Class 3 - Social representations involved in the meanings and feelings of conflicts since the discovery of the diagnosis; Class 4 - The importance of support networks in living with HIV; Class 5 - Challenges in prevention and sexuality among women with HIV.

### Class 1 - Social representations of aging with HIV

In this category, the reports demonstrate the similarity between living with the virus and the limitations naturally imposed by aging, evidenced by the recurrence of terms such as “age,” “to age,” “elderly,” “to limit,” and “to live”.

*What do I think about having HIV? It’s something I haven’t accepted yet, much less reaching this age living with it.* (W1, 64 years old, diagnosed more than 20 years ago)

[...] *me growing old with HIV? What worries me is what I think about in the future. When I can’t do things myself and have to have someone else do things for me.* (W2, 65 years old, diagnosed more than 20 years ago)

Therefore, it is not enough to simply exist under the perspective of prejudice and stigma surrounding the virus, but also under the misfortunes that the social imagination carries regarding the difficulties experienced by older adults.

*For me, it’s worse to be an older woman with this. I can’t accept it. I go this way, I take the bus, but every moment I have this on my mind* [...]. (W14, 51 years old, more than 20 years since diagnosis)

*When I think about being an older adults, because I am getting old while living with this disease, I think it’s more difficult because, in addition to HIV, other diseases also appear, and certainly, being an older adult with this is more difficult.* (W15, 50 years old, more than 20 years since diagnosis)

### Class 2 - Social representations involved in the meanings and feelings of adherence to antiretroviral therapies

In this category, as in other national surveys on the topic, it is common for respondents to comment on the timing and impact of discovering they were living with the virus.

*Because, for me, the diagnosis was a real shock. I thought that anyone who had this disease and found out about it would die the next day. That was my mindset.* (W3, 53 years old, more than 20 years since diagnosis)

*My feeling is one of outrage, because I was infected in my own home. I never had a promiscuous life, and I live with HIV without ever having had a promiscuous life, without ever having lived and enjoyed life.* (W26, 63 years old, more than 20 years since diagnosis)

*Oh my, I don’t even like to remember the diagnosis. HIV ruined years of my life, it took my whole life away.* (W22, 57 years old, time since diagnosis between 10 and 19 years).

### Class 3 - Social representations involved in the meanings and feelings of conflicts since the discovery of the diagnosis

Reinterpreting traumas and adverse reactions is one of the main challenges in healthcare services to ensure accurate adherence to ART. This is because it is crucial, first and foremost, to generate or encourage the recollection of positive memories to overcome limiting beliefs about living with, and especially aging with, the virus.

*I take the medication indiscriminately only because I need to keep living. Even after 16 years, I still feel disgusted taking these medications. When I see the medication, I already feel like vomiting. I usually take it with boldo tea and juice, but even today, I have difficulty.* (W18, 72 years old, time since diagnosis 10 to 19 years ago)

*I take my medication correctly now. There was only one time, a few years ago, when I stopped taking it. And I developed tuberculosis because I had stopped taking it, because the worst thing about that disease is those medications.* (W15, 50 years old, more than 20 years since diagnosis)

*But even today, I’m still in that fear, I keep looking around, and I think everyone is watching me and knows what I have. And that changes who you are* [...]*. That’s what kills me. I can’t talk about things; I keep hiding that I take these medications. And the problem isn’t even the HIV, it’s the consequences.* (W1, 64 years old, more than 20 years since diagnosis)

### Class 4 - The importance of support networks in living with HIV

In this class, we see how social relationships positively influence the way we think and act when living with HIV. Strategies such as group discussions with professionals and patients, family support, and help from institutions are useful tools. These institutions can be religious, educational, or focused on women’s empowerment, and they directly help these women cope with the experience and aging with HIV.

*My only fear was my father, who was alive at the time. But, to my surprise, he was the one who gave me total support* [...]*. My father and I took my eldest daughter at the time to a psychologist, and today she is the one who gives me the strength to continue.* (W27, 62 years old, more than 20 years since diagnosis)

*I only hear professionals from here and other leading hospitals talking about it, because where I live, even the professionals there don’t come close* [...]*. Because I’ve worked in healthcare and everyone makes a professional pact to care in a humane and unprejudiced way, but it’s no use, I’ve seen it in many places and even on television, and it only makes things worse.* (W25, 56 years old, time since diagnosis 10 to 19 years)

### Class 5 - Challenges in prevention and sexuality among women with HIV

The main difficulties these women face are related to the idea that prevention is limited to the use of condoms. Other factors in Brazilian culture hinder infection reduction. Patriarchy, gender inequalities, shared responsibility in marital relationships, and lack of information about transmission are examples.

*So, we didn’t use condoms because he was my husband and I had nothing to fear. I thought I was his only one, and that was my mistake, because while I was his only one, he wasn’t only mine.* (W17, 52 years old, time since diagnosis 10 to 19 years ago)

*I think people only get it through sexual relations; I don’t know if it’s through hugging or other ways. I only know that I was only in contact with him for a month, and he already had it. I always used condoms, except with him because he didn’t like them. He didn’t want to use them, and when he did, it was reluctantly.* (W13, 59 years old, time since diagnosis 6 to 11 months)

*Nowadays, things have improved a bit. People are starting to talk about the ways of getting infected, like through injections or tattoos. I myself rarely used condoms and got infected; I don’t know if it was through drug use or sexual intercourse. The doctor said it was through intercourse.* (W11, 69 years old, more than 20 years since diagnosis)

## DISCUSSION

The narratives of the 30 women studied were influenced by their experiences over time, from diagnosis to the moment of the interview. The information reveals distinct views about the aging process with HIV, considering it as a process and a phenomenon that is often negative.

Many of these women were influenced throughout their lives by the information presented in the diagnostic images of their illness. They were also affected by attitudes and concepts about collective and individual subjectivity regarding the order, morality, and spirituality of human beings, directly impacting the process and shaping common opinions about life and health^(15,16)^.

This is particularly significant for women who have HIV or have had AIDS for a long time and are in advanced stages of life, having lived through more than half of the AIDS trajectory in the country and the world in the last 40 years. The meanings found in this research allowed us to highlight a central theme that these women express when portraying HIV: the experience of and aging with HIV is sad, painful, and revolting^(16-18)^.

From the perspective of SRs according to Jodelet^(13)^, social norms shape women according to established patterns of sexuality, body, age and health. Even individually, women tend to go beyond the social, trying to make sense of and reinterpret the experience of living and aging with HIV, although these thoughts are not consensual.

However, most narratives express themselves as a way of embracing and caring for their own changes. They go far beyond the stigmas and prejudices of the social imagination regarding these issues. Thus, SRs are grounded in categories that encompass discovery, the several elements involved in treatment, and aging with HIV/AIDS^(17)^.

This occurs because, throughout their lives, these women develop values, beliefs, patterns, and evaluative status regarding the phenomenon of health/illness^(18)^. Classes 1, 2, and 3 help in understanding the general dimensions of living and aging with HIV. On the other hand, classes 4 and 5 justify the development of practices and activities based on the collaboration or lack thereof with support strategies.

The first three classes offer a general overview of the national and international situation experienced by developing countries in relation to gender issues. This scenario is strongly rooted in the memories of these women, reflecting the oppression and interaction with the moralistic beliefs and values that permeate individuals and society^(17-20)^.

Therefore, living and aging with HIV, despite the stigmas and prejudices, is not the main difficulty. It is also necessary to give voice to these women silenced for many years in the fight against AIDS worldwide. Many of them have been silenced throughout history, fighting for the right to their own bodies and chances at life, in a process of feminization not only of an epidemic, but of authorship over their own history and quality of life^(20)^.

Unequal sociopolitical and economic circumstances, such as those experienced between gender, race, and ethnicity, contribute to the development of ambiguous feelings. Although not the primary cause, it can be a relevant factor in vulnerability to sexually transmitted infections, affecting not only diagnosis but also living with the virus^(18-22)^.

The representational content present in this research is cruel, guiding actions and behaviors. It generates taboos, denies the state of health, hinders adherence to treatment, and delays a positive perception of aging with HIV, as evidenced by terms such as “life”, “age”, HIV, “cure”, “little”, “knowledge”, “health”, and “live”.

When recalling past events, these women develop a negative view of life and aging due to the virus. Understanding HIV/AIDS treatment is supported by several factors that sometimes cause confusion. These factors include well-being, adherence to treatment, and the positive and stable evolution of the infection. This contributes to the aging process associated with HIV^(23)^.

According to Moscovici^(5)^, individuals’ or groups’ behaviors are affected not only by the characteristics of situations, but also by the SRs of those situations. Jodelet^(13)^, in her study, shows that SRs guide people’s behavior towards patients. In accordance with the approach used by Jodelet, this study suggests that social practices can impact SRs in various situations, including unprotected sex, dignity, lack of knowledge about modes of transmission, perception of invulnerability, and correct use of ART. These are linked to several factors, such as economic, social, and educational inequalities, and a status quo of stable, lasting, and morally correct love^(18-25)^.

One of the main challenges in preventing and controlling AIDS and HIV is the regular use of medication and sexual protection. This study observed practices related to privacy and confidentiality of treatment often associated with stigma and discrimination. The effectiveness of support networks is hampered by the constant presence of stigmatizing information about these groups^(3,24)^.

In each participant’s account, several mentions can be seen of the experience of women aging with HIV. Aging is, therefore, a difficult process, with various changes in social, professional, and family life, as well as in social activities^(25)^.

This study did not focus on the importance of HIV/AIDS treatment based on the decisions of the women interviewed, nor did it assess the use of antiretroviral medications. The success of therapy depends on several aspects, including patients’ commitment to the regimen and its persistence. Another aspect frequently mentioned in the reports of group 4 was the importance of family, professional, and spiritual support^(23-28)^.

It is essential to transform the models and methods of health prevention in the country. Weaknesses in HIV/AIDS prevention and continuing care within healthcare services were highlighted among the participants in this study, particularly in the context of Primary Care^(25)^. Strengthening this level of healthcare within the Healthcare Network should be a focus of discussions at all levels of government across the country^(24-28)^.

However, it is important to strengthen social participation in decisions related to the country’s health. Encouraging women to assume leadership positions in various contexts, such as group discussions, health projects, and communities, is a possible strategy. This promotes equality through community actions that offer equal opportunities, respect, and well-being^(23-28)^.

Finally, the most relevant contribution of this theory to studies on coping with AIDS and HIV is to provide changes, focused on long-term strategies, that can modify the SRs passed on to future generations, since these are mutable. Is it a utopia? Perhaps, but by creating new symbolic and interpretative structures that positively influence emotions and social behaviors, it is possible to generate a new social identity based on new established customs and values that significantly contribute to the reduction of infections.

### Study limitations

The limitations of this study relate to the particularities of the qualitative approach and the use of IRaMuTeQ. Because it is qualitative research, the results cannot be generalized, as they reflect perceptions situated in a specific context. The use of IRaMuTeQ, while useful for lexical analysis, presents weaknesses such as dependence on the text *corpus’s* consistency, the possibility of semantic losses in categorization, and the need for interpretation by the researcher, which can introduce biases. Furthermore, the investigation was conducted in only two SAEs, which restricts the diversity of perspectives considered.

### Contributions to nursing, health, or public policy

This study may contribute to a critical reflection on HIV/AIDS prevention and control practices and actions in the female population, with a special focus on women over 50 years of age. In this way, it can serve as a source of data for future studies, assisting in the structuring and implementation of strategies for multidisciplinary care.

## FINAL CONSIDERATIONS

This study analyzed how women with HIV who receive care at SAEs view their lives after diagnosis and aging with the virus, considering daily events, practices, and activities related to this condition, seeking to understand the SRs involved in this process.

Based on this data, it was observed that participants experienced emotional changes in relation to HIV, influenced by the intensity of their lives and their experience with the disease, caused by factors related to prejudice, stigma, concepts, and images associated with the virus in the past and in their daily lives with the virus.

This study highlighted the importance of a structured support network in generating positive feelings about coping with life, adhering to ART, and the self-perception of aging with the virus. Family, friends, healthcare professionals, and places to practice spirituality are examples of this.

It also revealed potential weaknesses in the continuing education of healthcare professionals and in healthcare services. The shift in healthcare paradigms must encompass not only qualification but also transformations in the macro and micro-policies of the country’s healthcare system, allowing for the provision of humanized and high-quality care that goes beyond current preventive and biomedical models.

Finally, it is important to consider gender-related socioeconomic vulnerabilities. In this study, this was one of the main reasons for the difficulties faced in perceiving one’s own health. Increased psychosocial support and community participation are suggested, using methods such as Integrative Community Therapy and Popular Education to strengthen and empower these women in their own lives and health.

## Data Availability

The research data are available within the article.
