# Supplementary material for: Between living and aging: perceptions of women with HIV
Source: Rev Bras Enferm. 2026 Jul 27;79:e20250084. doi: 10.1590/0034-7167-2025-0084 (PMC13411318; doi:10.1590/0034-7167-2025-0084)
Supplement: Translation

# Translation: Entre viver e envelhecer: percepções de mulheres com HIV

**DOI:** 10.1590/0034-7167-2025-0084pt

**Authors:** Evanilza Maria Marcelino, Jaciel Bezerra da Silva, Carla Andreia Alves de Andrade, Sergio Corrêa Marques, Aurélio Molina da Costa, Denize Cristina de Oliveira, Fátima Maria da Silva Abrão

**Affiliations:** I Universidade de Pernambuco. Recife, Pernambuco, Brasil; II Universidade Federal de Alagoas. Maceió, Alagoas, Brasil; III Universidade do Estado do Rio de Janeiro. Rio de Janeiro, Rio de Janeiro, Brasil

**Keywords:** Representação Social, Mulheres, Envelhecimento Saudável, HIV, Enfermagem.

## Abstract

**Objetivos:**

analisar as percepções do viver e do envelhecer entre mulheres com HIV.

**Métodos:**

pesquisa qualitativa com 30 mulheres com idade igual ou superior a 50 anos em Recife, PE, Brasil. Utilizaram-se a Teoria das Representações Sociais, como referencial teórico, e o método de análise, lexical através do *software Interface de R pour les Analyses Multidimensionnelles de Textes et de Questionnaires*.

**Resultados:**

a representação social do envelhecer com HIV é vista sob a perspectiva temporal de uma trajetória de vida triste, dolorosa e revoltante, determinando significados sobre o diagnóstico, as práticas e atividades de vida diária, conflitos nas relações humanas, e adesão a terapias antirretrovirais.

**Considerações Finais:**

a importância dessas representações remete à melhoria da prática dos profissionais de saúde e promove uma compreensão mais ampla do envelhecer com HIV, oferecendo cuidado mais humanizado, integral/dinâmico nos serviços de saúde, e considerando a qualidade de vida em diferentes contextos sociais.

## INTRODUÇÃO

O tempo passa, mas os construtos do pensamento social permanecem transitórios e variáveis nos 40 anos desde a descoberta da Aids no Brasil. Com o sucesso das terapias antirretrovirais (TARVs), apesar de aumentar a expectativa/qualidade de vida e reduzir os casos de Aids, as infecções pelo Vírus da Imunodeficiência Humana (HIV) não têm seguido essa proporção^(1)^.

Em 2021, o panorama mundial e nacional foi preocupante, com 37,7 milhões de pessoas vivendo com HIV no mundo, dos quais 53% são mulheres. Houve um aumento na proporção de casos de HIV entre mulheres com 50 anos ou mais de 12,2%, em 2011, para 17,9%, em 2021^(1,2)^.

Com o passar dos anos, as pessoas que contraíram o vírus HIV têm maior probabilidade de contrair doenças relacionadas à idade, implicando diretamente os indicadores de saúde e a autopercepção positiva diante do viver e envelhecer com o vírus^(2,3)^.

A Teoria das Representações Sociais (TRS) é relevante para entender fenômenos sociais. Ela se propõe a reconhecer e amplificar a compreensão do senso comum, podendo interferir no comportamento dos indivíduos. Moscovici, com sua teoria, desenvolveu processos que facilitam a troca de informações e a produção coletiva de conhecimento em comunidades em relação a um objeto comum^(4,5)^.

Segundo Jodelet, as representações sociais (RSs) interpretam a realidade cotidiana com base em conhecimentos compartilhados entre indivíduos e grupos. Elas tornam familiar o novo e o incomum em seu universo representacional, dando sentido aos comportamentos adotados. No início da descoberta da Aids, os elementos visuais das RSs eram especialmente enfatizados nas propagandas da época. Isso gerou vários significados sociais, que hoje são confrontados com representações imagéticas de outros objetos sociais, como o envelhecimento feminino^(4-7)^.

As três dimensões presentes nas RSs permitem uma análise dimensional: a informação (conceito); o campo de representação (imagem); e a atitude. Neste estudo, o conceito é formado pela organização do conhecimento dos sujeitos sociais e do objeto social, variando conforme o tempo, quantidade e qualidade do conhecimento propagado^(8)^.

Refletindo sobre a subjetividade manifesta nas ações e reações ao diagnóstico, viver e envelhecer com HIV, as mulheres evidenciam RSs em seus discursos. Essas RSs são compreendidas como construções influenciadas ao longo do tempo por origens históricas, culturais, tradições, imagens, conceitos, atitudes e comportamentos^(5-8)^.

Este estudo é relevante, tendo em vista as lacunas encontradas no levantamento do estado da arte sobre o objeto estudado e sobre o grupo social escolhido para análise. A produção da TRS sobre HIV/Aids é pouco discutida em literaturas nacionais e internacionais. No entanto, é muito prática, diante das ações e dos comportamentos ligados à vivência com o HIV. Essa situação impacta significativamente as questões de gênero, saúde, economia e qualidade de vida^(9-12)^.

Pretende-se, com este estudo, entender mais sobre o tema e considerar o contexto dessas mulheres, propondo estratégias para lidar com a vida e o envelhecimento com o vírus.

## OBJETIVOS

Analisar as percepções do viver e do envelhecer entre mulheres com HIV.

## MÉTODOS

### Aspectos éticos

Foram observadas as normas estabelecidas de acordo com a Resolução nº 466/2012, que dispõe sobre as diretrizes e regulamentações para pesquisas envolvendo seres humanos, com aprovação concedida por Comitê de Ética em Pesquisa. As participantes foram esclarecidas por meio do Termo de Consentimento Livre e Esclarecido, sendo este posteriormente assinado por extenso ou por meio da assinatura digital do polegar direito.

### Referencial teórico-metodológico

Esta pesquisa foi fundamentada na abordagem processual das RSs desenvolvida por Denise Jodelet em seu estudo sobre as RSs da loucura^(13)^. As RSs são entendidas pela autora como um conhecimento socialmente elaborado e compartilhado, com um objetivo prático, que contribui para construção de uma realidade comum a um grupo social^(6)^.

A análise dos depoimentos foi realizada por meio do *software Interface de R pour les Analyses Multidimensionnelles de Textes et de Questionnaires* (IRaMuTeQ), ferramenta que possibilita o processamento e a organização de dados textuais de forma sistemática. Esse recurso permite identificar a frequência das palavras, estabelecer associações e classificar o material em categorias, favorecendo a compreensão das regularidades e especificidades presentes nos discursos^(14)^.

Aplicando essa perspectiva na enfermagem, especialmente no cuidado de mulheres idosas que vivem com HIV, as RSs se tornam fundamentais para compreender como essas mulheres percebem a si mesmas, a doença e o tratamento. As RSs influenciam diretamente questões como adesão à TARV, busca por apoio social, enfrentamento do estigma e a forma como lidam com o envelhecimento associado à infecção pelo HIV.

Além disso, ao estudar essas representações, os profissionais de enfermagem podem identificar barreiras simbólicas e culturais que impactam o cuidado, e elaborar estratégias mais eficazes e humanizadas. Isso também contribui para um atendimento mais sensível às especificidades dessa população, promovendo uma prática mais crítica, reflexiva e centrada na pessoa.

### Tipo de estudo

Trata-se de estudo exploratório-descritivo, com abordagem qualitativa. Foram realizadas entrevistas semiestruturadas, incentivando as pacientes a compartilhar suas percepções sobre o viver e envelhecer com HIV. O guia adotado para elaboração do relatório deste estudo foi o *COnsolidated criteria for REporting Qualitative research.*

### Cenário do estudo

Este estudo foi desenvolvido em dois Serviços de Assistência Especializada (SAEs) em HIV/Aids localizados em hospitais públicos na Região Metropolitana de Recife, Pernambuco.

### População

Participaram do estudo 30 mulheres escolhidas de forma aleatória, porém intencional. Todas são heterossexuais, com 50 anos ou mais, acompanhadas em dois SAEs. Além disso, possuíam diagnóstico de HIV há, pelo menos, seis meses. Mulheres com diminuição cognitiva ou distúrbios mentais foram excluídas desta pesquisa.

### Coleta e organização dos dados

A coleta de dados ocorreu de junho a agosto de 2023, por meio de um questionário sociodemográfico e clínico, além de um roteiro semiestruturado de entrevista para entender a subjetividade das participantes. Os temas do roteiro foram dirigidos ao campo das representações, às imagens e aos conhecimentos construídos pela população sobre o HIV, com o intuito de perceber as atitudes e a lógica das relações sociais em relação ao gênero e envelhecimento.

As entrevistas, com duração média de 11 minutos e 12 segundos, foram gravadas em áudio com a permissão das participantes, depois transcritas e preparadas para análise. Com objetivo de preservar a identidade destas, optou-se por identificá-las com a letra “M”, oriunda da palavra Mulher, junto ao número sequencial da entrevista, idade e tempo de diagnóstico (exemplo: M1, idade, tempo de diagnóstico há mais de 20 anos).

### Análise dos dados

Os dados de caracterização sociodemográfica e clínica foram consolidados no *software Microsoft Excel^®^* e analisados mediante estatística descritiva. Após a organização do banco de dados textual, o mesmo foi introduzido e processado com o apoio do *software* IRaMuTeQ, que forneceu uma descrição do conteúdo dos depoimentos das pesquisadas. Este *software* tem sido utilizado em estudos de abordagem qualitativa, principalmente em estudos da TRS, por fornecer técnicas estatísticas para auxiliar os pesquisadores a compreender o objeto social e criar classes de análise com base na identificação das unidades de significado^(14)^.

Entre os possíveis métodos de análise do *software*, optou-se usar o método Classificação Hierárquica Descendente, em que cada texto foi avaliado pelo método de classificação de material, fragmentado em partes e agrupados de acordo com as palavras para construir categorias.

### Declaração de IA generativa e tecnologias assistidas por IA no processo de redação

Durante a preparação deste manuscrito, foi utilizado o ChatGPT para auxílio na revisão gramatical e ortográfica, bem como na geração de sugestões para reformulação de trechos do texto. Após o uso desta ferramenta, o conteúdo foi revisado e editado conforme necessário, sendo assumida total responsabilidade pelo conteúdo final da publicação.

## RESULTADOS

Participaram do estudo mulheres heterossexuais, com a maioria (56,7%) possuindo idade entre 50 e 59 anos, e 46,7% vivendo com HIV há mais de 20 anos. A maioria (60%) se identifica como parda e tem ensino fundamental incompleto (56,7%). Quanto ao estado civil, 36,7% são casadas e 36,7% são solteiras. Há igual proporção de (40%) entre aposentadas e domésticas, sendo 60% delas católicas.

O *corpus* foi dividido em 669 segmentos de texto, constituídos pelo *corpus*. Desses, 586 segmentos de texto foram processados, e após diversas clivagens, estabilizaram e geraram cinco classes lexicais, com aproveitamento de 87,59%. Esse percentual representa um bom aproveitamento do material, uma vez que, de acordo com a literatura, é necessária a retenção mínima de 75% dos segmentos textuais fornecidos^(14)^.

As classes foram definidas conforme a Figura 1, sendo: Classe 1 - As representações sociais do envelhecer com HIV; Classe 2 - As representações sociais envolvidas nos significados e sentimentos na adesão às terapias antirretrovirais; Classe 3 - As representações sociais envolvidas nos significados e sentimentos nos conflitos desde a descoberta do diagnóstico; Classe 4 - A importância das redes de apoio diante do viver com HIV; Classe 5 - Desafios na prevenção e sexualidade entre mulheres com HIV.

**Figura 1:** Dendrograma e Classificação Hierárquica Descendente das classes geradas pelo IRaMuTeQ, Recife, Pernambuco, Brasil, 2025

### Classe 1 - As representações sociais do envelhecer com HIV

Nesta classe, os relatos demonstram a similaridade existente entre o viver com o vírus e as limitações impostas de forma natural pelo envelhecimento, evidenciada pela recorrência de termos como “idade”, “envelhecer”, “idoso”, “limitar” e “viver”.

*O que eu penso em ter HIV? É uma coisa que eu não aceitei ainda, muito menos chegar nessa idade vivendo com isso.* (M1, 64 anos, tempo de diagnóstico há mais de 20 anos)

[...] *eu envelhecendo com HIV? O que eu penso lá na frente é o que me preocupa. Quando eu não puder fazer as minhas coisas e ter que ter alguém para fazer as coisas por mim.* (M2, 65 anos, tempo de diagnóstico há mais de 20 anos)

Portanto, não é suficiente apenas existir sob a perspectiva do preconceito e do estigma do vírus, mas também sob os infortúnios que o imaginário social carrega a respeito das dificuldades experimentadas pela terceira idade.

*Para mim, é pior ser mulher idosa com isso. Eu não aceito. Eu vou por aqui, pego ônibus, mas a cada momento eu fico com isso na minha mente* [...]. (M14, 51 anos, tempo de diagnóstico há mais de 20 anos)

*Quando eu penso em ser idosa, porque eu estou ficando idosa vivendo com essa doença, penso que é mais difícil, porque além do HIV, vão aparecendo outras doenças também e, com certeza, ser idosa com isso é mais difícil.* (M15, 50 anos, tempo de diagnóstico há mais de 20 anos)

### Classe 2 - As representações sociais envolvidas nos significados e sentimentos na adesão às terapias antirretrovirais

Nesta categoria, assim como em outras pesquisas nacionais sobre o tema, é comum os entrevistados comentarem o momento e o impacto na descoberta do viver com o vírus.

*Porque, para mim, o diagnóstico foi um banho de água fria. Para mim, quem tinha essa doença e descobria logo morria no outro dia. Esse era meu pensamento.* (M3, 53 anos, tempo de diagnóstico há mais de 20 anos)

*O sentimento que eu tenho é de revolta, porque eu fui contaminada na minha casa. Eu nunca tive uma vida promíscua e vivo com HIV sem nunca ter tido uma vida promíscua sem viver e curtir.* (M26, 63 anos, tempo de diagnóstico há mais de 20 anos)

*Nossa, não gosto nem de lembrar do diagnóstico. O HIV acabou com os anos da minha vida, levou toda a minha vida embora.* (M22, 57 anos, tempo de diagnóstico entre 10 a 19 anos)

### Classe 3 - As representações sociais envolvidas nos significados e sentimentos nos conflitos desde a descoberta do diagnóstico

A ressignificação de traumas e reações adversas é uma das principais dificuldades nos serviços de saúde para garantir a adesão precisa das TARVs. Isto porque, é importante antes de tudo gerar ou estimular a lembrança de memórias positivas para superar crenças limitantes acerca do viver e, principalmente, envelhecer com o vírus.

*Eu tomo o remédio a pulso só porque eu preciso continuar a viver. Mesmo depois de 16 anos, eu ainda tenho nojo de tomar essas medicações. Quando eu vejo as medicações, já me dá vontade de vomitar. Eu tomo mais com chá de boldo e suco, mas até hoje, eu tenho dificuldade.* (M18, 72 anos, tempo diagnóstico de 10 a 19 anos)

*Eu tomo hoje a medicação direito. Só teve uma época, uns anos atrás, que parei de tomar as medicações. E eu desenvolvi tuberculose por conta que eu tinha parado de tomar, porque o ruim dessa doença é só essas medicações.* (M15, 50 anos, tempo de diagnóstico há mais de 20 anos)

*Mas até hoje, eu fico naquele temor, fico olhando, e acho que todo mundo está me vendo e sabendo o que tenho. E isso muda quem você é* [...]. *É isso que me mata. Eu não consigo contar as coisas, fico escondendo que tomo essas medicações. E o problema não é nem o HIV, é as consequências.* (M1, 64 anos, tempo de diagnóstico há mais de 20 anos)

### Classe 4 - A importância das redes de apoio diante do viver com HIV

Nesta classe, vemos como as relações sociais influenciam positivamente a forma de pensar e agir ao viver com HIV. Estratégias como discussões em grupo com profissionais e pacientes, apoio da família, e ajuda de instituições são ferramentas úteis. Essas instituições podem ser religiosas, educacionais ou focadas no empoderamento feminino, e ajudam diretamente essas mulheres a lidar com a experiência e o envelhecimento com HIV.

*Meu único medo era o meu pai, que era vivo na época. Mas, para minha surpresa, foi quem me deu total assistência* [...]. *Levamos, eu e meu pai, minha filha mais velha na época para uma psicóloga, e hoje é ela quem me dá forças para continuar.* (M27, 62 anos, tempo de diagnóstico há mais de 20 anos)

*Só vejo os profissionais daqui e dos outros hospitais de referência falar, porque, no local onde eu moro, mesmo os profissionais de lá não chegam nem perto* [...]. *Porque eu já trabalhei na saúde e todo mundo faz um pacto de profissão de cuidar de forma humanizada e sem preconceito, mas não tem jeito, eu já vi em muito canto e na televisão mesmo e isso só piora as coisas.* (M25, 56 anos, tempo diagnóstico de 10 a 19 anos)

### Classe 5 - Desafios na prevenção e sexualidade entre mulheres com HIV

As principais dificuldades dessas mulheres estão relacionadas à ideia de que a prevenção se limita ao uso de preservativos. Outros fatores da cultura brasileira dificultam a redução das infecções. O patriarcado, desigualdades de gênero, responsabilidade compartilhada em relações conjugais e falta de informação sobre transmissão são exemplos.

*Então, a gente não usava camisinha, porque ele era meu marido e eu não tinha o que temer. Eu achava que era a única dele, e esse foi meu erro, porque enquanto eu era a única dele, ele não era só meu.* (M17, 52 anos, tempo diagnóstico de 10 a 19 anos)

*Eu acho que as pessoas só pegam nas relações, não sei se pega abraçando e de outras formas. Só sei que só tive contato com ele um mês, e ele já estava com isso. Eu sempre usei camisinha, só com ele que não usei porque ele não gostava. Ele não queria usar, e quando usava, era a pulso.* (M13, 59 anos, tempo de diagnóstico de 6 a 11 meses)

*Hoje em dia, melhorou um pouco. As pessoas já começam a conversar sobre as formas de contaminação, como agulha injetável ou tatuagem. Eu mesma usei muito pouco camisinha e me infectei não sei se usando droga ou na relação sexual. O médico disse que foi na relação.* (M11, 69 anos, tempo de diagnóstico há mais de 20 anos)

## DISCUSSÃO

Os discursos das 30 mulheres estudadas foram influenciados pelas vivências no decorrer do tempo, desde o diagnóstico até o momento da entrevista. As informações evidenciam visões distintas acerca do processo de envelhecimento com HIV, considerando-o como um processo e um fenômeno muitas vezes negativo.

Boa parte dessas mulheres foi influenciada durante a vida pelas expressões nas informações da imagem diagnóstica da doença. Também interferidas pelas atitudes e conceitos sobre a subjetividade coletiva e individual acerca da ordem, moral e espiritualidade do ser humano, afetando diretamente o processo, e formando opiniões comuns sobre a vida e a saúde^(15,16)^.

Isso é particularmente significativo para mulheres que têm HIV ou tiveram Aids há muito tempo e estão em estágios avançados da vida, tendo vivido mais da metade da trajetória da Aids no país e no mundo nos últimos 40 anos. Os significados encontrados nesta pesquisa permitiram destacar um tema central que essas mulheres expressam ao retratar o HIV: a experiência e o envelhecimento com o HIV são tristes, dolorosos e revoltantes^(16-18)^.

Do ponto de vista das RSs, segundo Jodelet^(13)^, as normas sociais moldam as mulheres conforme padrões estabelecidos de sexualidade, corpo, idade e saúde. Mesmo individualmente, a mulher tende a ir além do social, tentando dar sentido e reinterpretar a experiência de viver e envelhecer com HIV, embora esses pensamentos não sejam consensuais.

No entanto, a maioria das narrativas se expressa como uma forma de acolher e cuidar de suas próprias mudanças. Elas vão muito além dos estigmas e preconceitos do imaginário social a respeito desses temas. Dessa forma, as RSs são fundamentadas em categorias, que abrangem a descoberta, os múltiplos elementos envolvidos no tratamento e o envelhecer com HIV/Aids^(17)^.

Isto ocorre porque, ao longo da vida, essas mulheres desenvolvem valores, crenças, padrões e *status* de avaliação sobre o fenômeno da saúde/doença^(18)^. As classes 1, 2 e 3 auxiliam na compreensão das dimensões gerais sobre o viver e envelhecer com HIV. Por outro lado, as classes 4 e 5 justificam o desenvolvimento das práticas e atividades a partir da colaboração ou não de estratégias de apoio.

As três primeiras classes oferecem um panorama geral da situação nacional e internacional vivenciada por países em desenvolvimento ligadas às questões de gênero. Esse cenário está fortemente enraizado nas memórias dessas mulheres, refletindo a opressão e a interação com as crenças e valores moralistas que permeiam o indivíduo e a sociedade^(17-20)^.

Para tanto, viver e envelhecer com HIV, apesar dos estigmas e preconceitos, não são a principal dificuldade. É necessário ainda dar voz a essas mulheres silenciadas por muitos anos de luta contra a Aids no mundo. Muitas dessas foram silenciadas ao longo da história, lutando pelo direito ao próprio corpo e chances de vida, em um processo de feminização não apenas de uma epidemia, mas de autoria sobre sua própria história e qualidade de vida^(20)^.

Circunstâncias sociopolíticas e econômicas desiguais, como as vivenciadas entre gênero, raça e etnia, colaboram para o desenvolvimento de sentimentos ambíguos. Embora não seja a causa principal, pode ser um fator relevante para vulnerabilidade às infecções sexualmente transmissíveis, afetando não apenas o diagnóstico, mas o viver com o vírus^(18-22)^.

Os conteúdos representacionais presentes nesta pesquisa são cruéis, orientando ações e comportamentos. Esses geram tabus, negam o estado de saúde, dificultam a adesão e retardam a percepção positiva do envelhecimento com o HIV presente nos discursos, evidenciados pelos termos “vida”, “idade”, HIV, “cura”, “pouco”, “conhecimento”, “saúde” e “viver”.

Ao relembrar eventos passados, essas mulheres desenvolvem uma visão negativa sobre a vida e o envelhecimento devido ao vírus. A compreensão do tratamento do HIV/Aids é apoiada por diversos fatores que, às vezes, causam certa confusão. Esses fatores incluem o bem-estar, a adesão ao tratamento, e a evolução positiva e estável da infecção. Isso favorece o processo de envelhecimento associado ao HIV^(23)^.

Para Moscovici^(5)^, os comportamentos dos indivíduos ou grupos são afetados não somente pelas características das situações, mas também pelas RSs dessas situações. Jodelet^(13)^, em seu estudo, mostra que as RSs guiam o comportamento das pessoas em relação aos doentes. Em conformidade com a abordagem utilizada por Jodelet, este estudo sugere que as práticas sociais podem impactar as RSs em várias situações, incluindo sexo desprotegido, dignidade, desconhecimento sobre modos de transmissão, percepção de invulnerabilidade e uso correto de TARV. Essas estão ligadas a diversos fatores, como desigualdades econômicas, sociais, educacionais, e a um *status quo* de amor estável, duradouro e moralmente correto^(18-25)^.

Um dos principais desafios para prevenir e controlar a Aids e o HIV é o uso regular de medicamentos e proteção sexual. Neste estudo, observaram-se práticas relacionadas à privacidade e confidencialidade do tratamento muitas vezes associadas ao estigma e discriminação. A eficácia das redes de suporte é prejudicada pela presença constante de informações estigmatizantes sobre os grupos^(3,24)^.

No relato de cada participante, podem-se ver várias menções à experiência de mulheres envelhecendo com HIV. O envelhecimento é, portanto, um processo difícil, com várias mudanças na vida social, profissional e familiar, assim como nas atividades sociais^(25)^.

Este estudo não focou na importância do tratamento do HIV/Aids com base nas decisões das mulheres entrevistadas, e não avaliou o uso dos medicamentos antirretrovirais. O sucesso da terapia depende de diversos aspectos, incluindo o comprometimento do paciente com o regime e a persistência deste. Outro aspecto bastante mencionado nos discursos da classe 4 foi a importância do apoio familiar, profissional e espiritual^(23-28)^.

É essencial transformar os modelos e métodos de prevenção de saúde no país. As fragilidades na prevenção e cuidado contínuo do HIV/Aids nos serviços de saúde foram destacadas entre as participantes deste estudo, principalmente no âmbito da Atenção Básica^(25)^. O fortalecimento deste nível de atenção em saúde dentro da Rede de Atenção à Saúde deve ser foco das discussões das esferas de gestão do país^(24-28)^.

Contudo, é importante fortalecer a participação social nas decisões relativas à saúde do país. Encorajar as mulheres a assumir posições de liderança em vários contextos, como discussões em grupo, projetos de saúde e comunidades, é uma possível estratégia. Isso promove igualdade através de ações comunitárias que oferecem oportunidades igualitárias, respeito e bem-estar^(23-28)^.

Por fim, a colaboração mais relevante desta teoria nos estudos de enfrentamento da Aids e HIV é proporcionar mudanças, focadas em estratégias de longo prazo, que podem modificar as RSs repassadas às próximas gerações, pois estas são mutáveis. É uma utopia? Talvez sim, porém, ao criar novas estruturas simbólicas e interpretativas que influenciem positivamente as emoções e comportamentos sociais, é possível gerar uma nova identidade social com base em novos costumes e valores estabelecidos que colaborem expressivamente na redução das infecções.

### Limitações do estudo

As limitações deste estudo se relacionam às particularidades da abordagem qualitativa e ao uso do *software* IRaMuTeQ. Por se tratar de pesquisa qualitativa, os resultados não podem ser generalizados, uma vez que refletem percepções situadas em um contexto específico. O emprego do IRaMuTeQ, embora útil para análise lexical, apresenta fragilidades como dependência da consistência do *corpus* textual, possibilidade de perdas semânticas na categorização e necessidade de interpretação pelo pesquisador, o que pode introduzir vieses. Ademais, a investigação foi realizada em apenas dois SAEs, o que restringe a diversidade de perspectivas contempladas.

### Contribuições para as áreas da enfermagem, saúde ou políticas públicas

Este estudo poderá contribuir para uma reflexão crítica sobre as práticas e ações de prevenção e controle do HIV/Aids na população feminina, tendo como foco especial as mulheres com mais de 50 anos. Dessa forma, pode ser fonte de dados para estudos futuros, auxiliando na estruturação e implementação de estratégias para o cuidado multidisciplinar.

## CONSIDERAÇÕES FINAIS

Este estudo analisou como as mulheres com HIV atendidas nos SAEs veem sua vida com o diagnóstico e o envelhecimento com o vírus, considerando os eventos diários, as práticas e atividades ligadas a essa condição, buscando a compreensão das RSs envolvidas neste processo.

A partir desses dados, percebeu-se que as participantes tiveram alterações emocionais em relação ao HIV, influenciadas pela intensidade da vida e pela experiência da doença, causadas por fatores relacionados ao preconceito, estigmas, conceitos e imagens relacionados ao vírus no passado e no convívio diário com o vírus.

Este estudo destacou a relevância de uma rede de apoio estruturada para gerar sentimentos positivos no enfrentamento da vida, na adesão às TARV e na própria autopercepção de envelhecimento com o vírus. Família, amigos, profissionais de saúde e locais para exercer espiritualidade são exemplos disso.

Revelou, também, possíveis fragilidades na formação contínua de profissionais de saúde e nos serviços de saúde. A mudança nos paradigmas de saúde deve abranger não apenas a qualificação, mas também transformações nas macros e micropolíticas da saúde do país, permitindo oferecer um atendimento humanizado e de qualidade que ultrapassa os modelos preventivos e biomédicos atuais.

Por fim, é importante considerar as vulnerabilidades socioeconômicas relacionadas ao gênero. Neste estudo, essa foi uma das principais razões para as dificuldades enfrentadas na percepção da própria saúde. Sugerem-se o aumento do suporte psicossocial e a participação na comunidade, usando métodos como a Terapia Comunitária Integrativa e a Educação Popular para fortalecer e empoderar essas mulheres em sua própria vida e saúde.

## Data Availability

Os dados de pesquisa estão disponíveis no corpo do artigo.
